# SCAMP: standardised, concentrated, additional macronutrients, parenteral nutrition in very preterm infants: a phase IV randomised, controlled exploratory study of macronutrient intake, growth and other aspects of neonatal care

**DOI:** 10.1186/1471-2431-11-53

**Published:** 2011-06-10

**Authors:** Colin Morgan, Shakeel Herwitker, Isam Badhawi, Anna Hart, Maw Tan, Kelly Mayes, Paul Newland, Mark A Turner

**Affiliations:** 1 Neonatal Intensive Care Unit, Liverpool Women's Hospital, Crown St, Liverpool L8 7SS, UK; 2 Department of Pharmacy, Aseptic Manufacturing Unit (MIA 12155), Royal Liverpool and Broadgreen University Hospitals NHS Trust, UK; 3 Department of Pharmacy, Liverpool Women's Hospital, Crown St, Liverpool L8 7SS, UK; 4 School of Health and Medicine, University of Lancaster, LA1 4YD, UK; 5 Community Child Health, Royal Liverpool Children's Hospital, Alder Hey, Liverpool L12 2AP, UK; 6 Dept Clinical Chemistry, Royal Liverpool Children's Hospital, Alder Hey, Liverpool L12 2AP, UK

## Abstract

**Background:**

Infants born <29 weeks gestation are at high risk of neurocognitive disability. Early postnatal growth failure, particularly head growth, is an important and potentially reversible risk factor for impaired neurodevelopmental outcome. Inadequate nutrition is a major factor in this postnatal growth failure, optimal protein and calorie (macronutrient) intakes are rarely achieved, especially in the first week. Infants <29 weeks are dependent on parenteral nutrition for the bulk of their nutrient needs for the first 2-3 weeks of life to allow gut adaptation to milk digestion. The prescription, formulation and administration of neonatal parenteral nutrition is critical to achieving optimal protein and calorie intake but has received little scientific evaluation. Current neonatal parenteral nutrition regimens often rely on individualised prescription to manage the labile, unpredictable biochemical and metabolic control characteristic of the early neonatal period. Individualised prescription frequently fails to translate into optimal macronutrient delivery. We have previously shown that a standardised, concentrated neonatal parenteral nutrition regimen can optimise macronutrient intake.

**Methods:**

We propose a single centre, randomised controlled exploratory trial of two standardised, concentrated neonatal parenteral nutrition regimens comparing a standard macronutrient content (maximum protein 2.8 g/kg/day; lipid 2.8 g/kg/day, dextrose 10%) with a higher macronutrient content (maximum protein 3.8 g/kg/day; lipid 3.8 g/kg/day, dextrose 12%) over the first 28 days of life. 150 infants 24-28 completed weeks gestation and birthweight <1200 g will be recruited. The primary outcome will be head growth velocity in the first 28 days of life. Secondary outcomes will include a) auxological data between birth and 36 weeks corrected gestational age b) actual macronutrient intake in first 28 days c) biomarkers of biochemical and metabolic tolerance d) infection biomarkers and other intravascular line complications e) incidence of major complications of prematurity including mortality f) neurodevelopmental outcome at 2 years corrected gestational age

**Trial registration:**

Current controlled trials: ISRCTN76597892; EudraCT Number: 2008-008899-14

## Background

The risk of significant neurocognitive disabilities in preterm survivors is well recognised, particularly under 26 weeks gestation [1,2]. Although many factors are associated with an increased risk of neurocognitive impairment, postnatal growth failure is now recognized as an important and potentially reversible risk [3-5]. Suboptimal growth is common in very low birthweight infants (VLBWI) [6,7] especially in those under 26 weeks [8]. Head growth is an especially important measure of growth failure because it correlates with brain growth [9]. Hack et al showed that subnormal head size at 8 months was predictive of poorer verbal and performance IQ scores at 3 [10] and 8 years [11]. Brain growth by 28 days after birth and the expected date of delivery are key predictors of long-term brain growth [12,13].

Early postnatal growth failure or extrauterine growth restriction describes the severe nutritional deficit that develops in preterm infants in the first few weeks of life [3,4]. The deficit refers to the gap between the energy and protein (and other nutrients) required to mimic fetal growth rates and the energy and protein that is actually delivered to the preterm infants. Current recommendations suggest a calorie intake of 120kcal/kg/day and a minimal protein intake of 2.5-3 g/kg/day. These are estimates based on matching fetal growth in utero [14] but do not take into account other factors that may increase individual infant requirements (such as catch-up growth, sepsis and chronic respiratory disease) and therefore increase the risk of postnatal growth failure [15]. Indeed, postnatal malnutrition may be inevitable based on current recommendations [16,17] and is exacerbated by huge variations in neonatal nutritional practice [18-21].

Very preterm infants have a gut that is too immature to digest milk in sufficient quantity to meet nutritional requirements. Virtually all preterm infants <29 weeks gestation and <1200 g require parenteral nutrition (PN) for a period that depends on gestation birthweight and other morbidities. The mean duration of PN (>75% all nutrition) in these infants (survivors) is 15.6 days [17] increasing to 20.8 days for infants <700 g [6]. Given these infants have the highest incidence of early and late growth failure and long term neurocognitive disability, effective PN delivery is essential to avoid major early nutritional deficits in these infants.

Inadequate and/or inconsistent nutritional strategies are one barrier to effective PN delivery but there are others. The most important is metabolic "intolerance". Early concerns about amino acid tolerance [22] continue to have profound effects on nutritional policies [23]. More recent evidence evaluating neonatal amino acid PN formulations, suggests amino acids can be rapidly introduced without metabolic complications [24-28] even in sick infants [29] and without causing acidosis [30]. This is essential if fetal protein accretion rates are to be matched and the large protein deficits which are routinely encountered in the first week of life are to be avoided [31]. Recommended maximum protein intake is 4 g/kg/day [31].

Optimal utilisation of protein for preterm infant growth depends on an adequate non-protein energy intake. A minimum of 20-25kcal/g protein is required [22,32] indicating that 100-120kcal/kg/day is needed to achieve maximal protein accretion [33]. Glucose and lipid infusion rates needed to achieve this may not be tolerated, especially in the first week, leading to hyperglycaemia and hyperlipidaemia. Increasing protein intake without providing an adequate non-protein calorie intake may result in growth failure and increased blood levels of urea and amino acids [34]. Carbohydrate may be the major determinant of optimal growth in preterm infants [35] and should account for 60-75% calories [31]. Glucose intolerance can be managed with reducing intake but is routinely managed effectively with an insulin infusion [36,37] although the long term risks and benefits of this approach are still unknown.

Postnatal growth can be improved with increased macronutrient intake [38-40] but evidence for an effect long-term neurodevelopment is more limited. Early introduction of amino acids [41] can also improve short term postnatal growth but in this study [41], persistent differences in head circumference did not translate into altered neurodevelopment outcome. Tan et al [17] did not show improved neurodevelopmental outcome with increased macronutrient intake but did not achieve the differences in nutritional intake expected. A correlation between protein and energy deficit (first 28 days) head growth at 36 weeks CGA was demonstrated and energy deficit (28 days) was associated with worse neurodevelopmental outcome at 3 months [42]. Early nutritional intake of a cohort of extremely low-birthweight survivors [43] has been correlated with 18 month neurodevelopmental outcomes. This suggested that early head growth failure may have a lasting effect on neurocognitive ability even if there was subsequent catch up growth before term. Provisional reports from other population-based cohort studies have supported this association [44] suggesting a change in head circumference z-score of -1.4 between birth and 28 days. This is consistent with our own audit findings and those of Tan et al (unpublished data) suggesting head growth failure reaches a nadir at approximately day 28. However, evidence linking early nutritional intervention with improved early head (and then ultimately neurodevelopmental outcome) is still lacking.

The final barriers to effective early nutrient delivery in the very preterm infant are PN prescription, formulation and administration. The conventional neonatal PN strategy has been based on individualised neonatal PN (iNPN) prescription and formulation to address the rapidly changing and variable fluid and electrolyte needs characteristic of the very preterm infant. This can be at the expense of early nutritional strategy when the evidence base supports early and consistent macronutrient delivery. Poor neonatal PN prescribing practice contributes to poor nutrition [45,46] and computer aided prescribing [47] can improve protein and energy intake [48,49]. However, iNPN has other limitations. Although iNPN prescription is flexible, the manufactured individualized PN bag is not so rapid responses to changes in fluid and electrolyte requirements after manufacture is not possible. When Tan et al [17] compared 2 iNPN regimens with a 30% difference in prescribed macronutrient content, the difference in actual energy and protein intake was <15%. This inefficiency in PN delivery was due to co-administration of other drug infusions, fluid restriction and changing electrolyte requirements. Thus, maximising nutritional intake in very preterm infants cannot be guaranteed by simply increasing the macronutrients in the PN formulation.

Standardising neonatal PN has been considered as an alternative to iNPN regimens [50] but has receive scant attention in published guidelines [31,51]. Early evidence suggested iNPN was required to meet the complex of the preterm infant [37]. Although some recent studies concur [49,52] increasing evidence suggests that with careful attention to local workload and PN prescribing practice most infants can be managed on a standard PN formulation [53-60] sometimes with improved macronutrient intake. Standardised PN solutions that allow some flexibility with electrolytes can overcome the variability in preterm electrolyte needs [56]. Increasing the concentration of neonatal PN (reducing the volume) has the potential to maintain nutritional intake in the face of fluid restriction and multiple drug infusions. Conventionally, stability and osmolality concerns have limited this approach, but current guidelines have virtually no evidence base. High osmolality of aqueous PN solutions can be offset by concurrent administration of intravenous lipids and dextrose.

Using the standardisation and concentration concepts, the preterm infant's competing needs for extreme flexibility for fluid and electrolyte management versus consistent optimal nutritional delivery can be accommodated in a "two compartment" PN model. We developed a standardised concentrated neonatal PN (scNPN) regimen that comprised a relatively inflexible (protected) nutrition compartment (85 ml/kg/day aqueous PN and 15 ml/kg/day intravenous lipid) and a highly flexible supplementary fluid compartment (usually 50 ml/kg/day). This supplementary compartment is then reduced or increased as total fluid requirements demand. Unexpected electrolyte derangement is corrected using standardised electrolyte infusions that replace part of the supplementary infusion as required. All standardized drug infusions are managed in the same way. Changes in infusion rate are titrated against the supplementary infusion not the nutrition compartment. Finally, early introduction of enteral feeds results in the reduction of the supplementary infusion until the enteral feed rate exceeds the supplementary infusion rate. Only then is PN reduced. This system allows maximum flexibility of fluid, electrolyte and drug infusion management with minimal impact on nutrient delivery.

We have shown the scNPN system of PN delivery is more effective at delivering protein, with >90% infants receiving >90% prescribed protein [60]. This lead to a 20% increase in the first 14 day protein intake when compared to a nutritionally identical iNPN regimen [60]. Significant cost reductions were also achieved (38%) similar to those reported for other standardised regimens [57]. There are no randomised controlled trials comparing standardised versus individualised neonatal PN, probably because logistics and patient safety considerations make this unfeasible in the complex very preterm population. However, given the potential benefits of the scNPN, a randomised controlled trial comparing the existing scNPN regimen with one where macronutrient content was maximised (scNPN_max_) is desirable.

### Hypothesis

We speculate that the scNPN and scNPN_max _regimens will provide efficient macronutrient delivery in the early neonatal period. We propose that optimising early protein and energy intake will partially correct early head growth failure characteristic of infants <29 weeks gestation. This could have implications for long term neurodevelopment. We hypothesise that the 30% increase in protein and calories achieved by the scNPN_max _regimen will lead to a significant improvement in head growth velocity over the first 28 days of life.

#### Primary objective

To compare the two allocation groups with respect to the rate of head growth from measurement made at enrolment to a measurement made between 27 and 29 completed days after birth (i.e. change in head circumference/(time of last measurement-time of first measurement)

#### Secondary objectives

To compare the two allocation groups with respect to the following:

a) growth measures ( 7, 14, 21 28 completed days and at 36 weeks corrected gestational age (CGA):

- occipitofrontal head circumference (OFC), weight, mid-upper arm circumference (MUAC) and lower leg length (LLL)

- modelling of weekly head growth, protein and calorie intake data

b) the efficiency of nutrient delivery (including protocol violations).

Nutritional intake at 7, 14, 21 and 28 days

- energy, protein, fat, glucose (including energy and protein deficits)

- predicted iNPN intakes based on mathematical model

c) the tolerance to each regimen by identifying abnormalities (and any required clinical interventions) in the following:

Nutritional tolerance (first 28 days or duration of PN):

- protein: daily serum urea, metabolic acidosis, amino acid profile day 7 and 21.

- fat: weekly triglyceride profile, hyperlipidaemia

- glucose: hypo/hyperglycaemia (including insulin use)

Biochemical tolerance (first 28 days or duration of PN):

- serum electrolytes, bone biochemistry and liver function

Use of supplementary electrolyte infusions

d) other recognised PN complications

Vascular access device usage and non-infective complications

- Vascular access device complications including extravasation injury

Infection:

- number of positive blood cultures

- number of infection and suspected infection episodes

e) Major neonatal morbidity

- Necrotising enterocolitis or focal intestinal perforation

- Chronic lung disease

- Intracranial abnormality on cranial ultrasound scan or other imaging

- Pulmonary haemorrhage

- Patent ductus arteriosus

- Retinal surgery

f) Neurodevelopmental outcome at 2 years (assessed using Bayley III scales)

## Methods/Design

### Trial design

A single centre, parallel group, randomised controlled trial with blinding of parents and outcome assessors. The control group will receive the standardised, concentrated neonatal parenteral nutrition formulation (scNPN) used in current clinical practice and the intervention group will receive a similar formulation containing additional macronutrients (scNPN_max_).

### Ethical and regulatory approval

Ethical approval was confirmed in May 2009 (09/H1008/91) by the Central Manchester REC (UK). Medicines and Healthcare products Regulatory Agency (MHRA) approval was given in May 2009.

### Inclusion criteria

Infants born 24^+0^-28^+6 ^weeks gestation who weigh <1200 g and who are admitted to the Neonatal Unit at Liverpool Women's Hospital within 48 hours of birth.

### Exclusion criteria

a) Infants who are unlikely to survive the first week after birth.

b) Infants diagnosed with major congenital or chromosomal abnormalities known to affect gastrointestinal function

c) Infants diagnosed with major congenital or chromosomal abnormalities known to affect head growth including definite parenchymal lesions on cranial ultrasound scan in first 48 hours.

d) Parents who are unable to give informed consent

### Eligibility and consent

Eligible patients will be identified from the electronic patient data management system by the Investigator. The parent/guardian(s) of each potentially eligible patient will be approached when the baby has achieved respiratory and haemodynamic stability, usually at approximately 48 hours. When clinical circumstances permit the parents of a potentially eligible baby will be approached before birth. The Investigator will explain the study fully to the patient's parent(s)/guardian(s) using the Patient Information Leaflet. The parents will have a minimum of 2 hours to consider the study but study information can be considered for a period up to 120 hours from birth.

### Randomisation

Where feasible, randomisation should occur before 72 hours of age where possible but must occur within 120 hours. Randomization codes will be computer generated using the statistical package STATA. Once generated the randomisation lists will be sealed in opaque serially numbered envelopes and given to pharmacy to store in a secure place. The randomisation list will be stratified by gestation at birth: 24-26 and 27-28 completed weeks gestation at birth. Once a patient is consented in to the trial, pharmacy will open the next sequential envelope in the correct strata and provide the allocated interventions. Allocation concealment will be maintained except in the Pharmacy Department at Liverpool Women's Hospital. In the case of multiple births, each infant will be individually randomised.

### Subject withdrawal

Patients may be withdrawn if the parent(s)/guardian(s) withdraws consent. Following withdrawal patients will be managed according to usual clinical practice. This means the patient will receive scNPN and routine biochemical and growth monitoring. Parents will be asked whether or not they consent to trial-related data to be collected for their baby(ies) and whether or not they consent to the continued use of information that has already been collected about their child.

Occasionally, infants on PN can become metabolically unstable (as determined by routine biochemical monitoring). This is usually managed by stopping or reducing PN and then gradually reintroducing PN once things improve. If such improvement is not sustained then an independent clinician and biochemist will discuss the need for possible withdrawal from the trial.

### Blinding

The manufacture and labelling of scNPN and scNPN_max _will take place at the Department of Pharmacy, Aseptic Manufacturing Unit, Royal Liverpool and Broadgreen University Hospitals NHS Trust (RLBUHT). This will allow Pharmacy at Liverpool Women's Hospital (LWH) to allocate the correct treatment according to randomisation while ensuring the final presentation of parenteral nutrition at the cotside will be in a form that does not reveal treatment allocation. Similarly, none of the prescription charts or documentation will indicate treatment allocation. This will effectively blind parents, most clinicians involved in patient care and individuals assessing study end-points. It will be possible for the prescriber (and the neonatal nurse or any other clinical person checking the prescription) to recognise different treatment allocations during the prescribing and administration process. This system ensures the safe prescription of PN using the existing robust supervisory framework. The Pharmacy Department at Liverpool Women's Hospital will record treatment allocation and will be able to "break the code" if a serious adverse event occurs, or at the request of the DMEC.

### Record of study participation

In accord with R&D policy at LWH, the notes of all participants will be marked with a sticker (notes) or a"tag" (electronic records). All clinical records of study participants will be retained for 20 years. All paper and electronic records relating to the study will be retained for 20 years.

## Methods: Treatment Regimen

### Study parenteral nutrition

Neonatal PN is manufactured under EU Good Manufacturing Practice at the Department of Pharmacy, Aseptic Manufacturing Unit, Royal Liverpool and Broadgreen University Hospitals NHS Trust (RLBUHT). The scNPN formulation is constituted according to the policy for Neonatal Parenteral Nutrition at Liverpool Women's Hospital the dispensing pharmacy will oversee the treatment allocation and the dispensing of study PN. The scNPN_max _is manufactured using the same policy guidance and differs only in the macronutrient content.

This study will compare two standardised concentrated neonatal PN regimens. The current standardised, concentrated formulation of PN (scNPN) together with a system of fluid and electrolyte management that allows effective nutritional delivery will comprise the control group. The intervention group will receive scNPN_max_. The scNPN_max _regimen follows the same administration protocol as the scNPN regimen but has a greater macronutrient content (Table 1). The other components of the scNPN_max _regimen are identical to that of scNPN. Thus, 3 nutritionally identical aqueous PN bags, MAX1 (no electrolytes), MAX2 (maintenance electrolytes for preterm infants) and MAX3 (MAX2 with additional sodium) cater for the different electrolyte requirements as described above for STD1, STD2 and STD3. The levels of macronutrient present in scNPN_max _fall within international recommendations [31] and are consistent with those studies providing the evidence for early, aggressive nutritional strategies [3,4].

**Table 1:** Comparison between scNPN and scNPN_max _macronutrient content and PN fluid volumes in a total fluid volume of 150 ml/kg/day

PN component	scNPN	**scNPN**_**max**_
Maximum protein (g/kg/day)	2.8	3.8
Maximum lipid (g/kg/day)	2.8	3.8
Maximum glucose (g/kg/day)	13.5	15.6
Total calorie intake (kcal/kg/day)	85	103
Maximum aqueous PN volume (ml/kg/day)	85	100
Maximum intravenous lipid volume (ml/kg/day)	15	20
Maximum supplementary dextrose volume (ml/kg/day)	50	30

### Description, labelling and storage of PN

The pharmacy at Liverpool Women's Hospital (LWH) and the Pharmacy Aseptic Manufacturing Unit at the RLBUHT will coordinate the provision of study scNPN and scNPN_max _to ensure there is sufficient and appropriate supply to all patients in the study. The pharmacy at Liverpool Women's Hospital and the Pharmacy Aseptic Manufacturing Unit at the RLBUHT will be responsible for tracking the allocation of all trial-related materials.

PN will be presented as:

a) a bag containing the aqueous PN components. During manufacture

- bags for the scNPN regimen will be labelled as STD1, STD2, STD3

- bags for the scNPN_max _regimen will be labelled as MAX1, MAX2, MAX3

b) a syringe containing intralipid

c) a syringe containing supplementary dextrose infusion

### Administration

The administration of scNPN (or scNPN_max_) will follow the current LWH NICU PN administration guidelines and will not differ from PN administration in infants not in the study (these infants will all receive scNPN). Following birth, scNPN will be administered until consent is obtained and the patient randomised to receive either scNPN or scNPN_max_. In accordance with the PN guidelines, PN administration will continue until the child is on 75% enteral feeds. If enteral feeds are stopped or markedly reduced (<25% total intake) after this point and the infant is <28 days, the original study PN will be restarted as soon as practical. If feeds are reduced but still exceed 25% total, study PN will be reintroduced only if enteral feeding <75% persists for more than 24 hours. All infants who need PN after 28 days will be prescribed scNPN. The introduction of PN, PN infusion rates (including the management of supplementary infusions) and reduction of PN with increased enteral feeds are described in detail in LWH NICU PN guidelines.

### Intolerance and over-dosage

The ability of individual infants to tolerate different PN components varies greatly, with age, gestation and clinical condition all contributing. This unpredictability requires regular and frequent biochemical monitoring described in LWH NICU PN guidelines. Clinicians and pharmacists will monitor PN tolerance and make necessary adjustments to PN administration as determined by daily clinical information and biochemical monitoring.

### Assessment of compliance with study PN

The amount prescribed is not necessarily the amount that a baby receives. Effectiveness of PN delivery is a secondary outcome for this study. Detailed and comprehensive information about the amount of PN infused is collected in the medical record. This will be transcribed to the CRF. This will allow accurate calculation of actual daily PN administration to individual patients. The results of these calculations will be recorded on the CRF. Expected daily PN is also recorded in the medical record. This will allow identification of any major deviation (>15 ml/kg/day) from the LWH guidelines. Non-trial PN will be administered until the infant is randomised. Following randomisation, administration of the non-trial PN/fluids may occasionally occur (eg severe hypoglycaemia, transfer to operating theatre or another centre). Administration of non-trial PN/fluids will still be fully recorded to allow full nutritional for the first 28 days to be calculated.

### Concomitant medications/treatments

These will be administered to all patients in accordance with the existing LWH PN guidelines and LWH NICU drug formulary. The study will not affect the use of concomitant medications/treatments.

## Methods: Assessments and Procedures

### Study schedule

The study schedule is summarised in Table 2. Randomisation will occur within 120 hours of birth. Following randomisation, baseline growth measurements will be performed. The study PN will be introduced at the earliest opportunity following randomisation. The process of collecting large amounts of routine monitoring data has been evaluated and refined in a previous study [51]

**Table 2:** Daily flow chart summarising PN administration (maximum possible) and data collection

	PN administration (macronutrient content)								Week	Data collection (nutrition)					
**Age (d)**	**Protein (g)**		**Lipid (g)**		**Dextrose; PN (%)**		**Dextrose; Suppl (%)**		**1**	**Enteral/IV fluid intake (ICR)**	**U/EBG**	**Bone/LFT**	**TG**	**AA**	**Growth**
							
	**std**	**max**	**std**	**max**	**std**	**max**	**std**	**max**	**PN type**						

**1**	1.8	1.8	1.0	1.0	10	10	10	10	PN	•	•				•

**2**	1.8	1.8	1.0	1.0	10	10	10	10	Consent &	•	•	•			

**3**	2.4	2.9	1.9	1.9	10	12	10	12	randomise	•	•				•

**4**	2.4	2.9	1.9	1.9	10	12	10	12		•	•	•			

**5**	2.8	3.8	2.8	2.8	10	12	10	12		•	•				

**6**	2.8	3.8	2.8	2.8	10	12	10	12	SCAMP	•	•	•			

**7**	2.8	3.8	2.8	3.8	10	12	10	12	SCAMP	•	•		•	•	

	**PN administration (macronutrient content)**								**Week**	**Data collection (nutrition)**					

**Age (d)**	Protein (g)		Lipid (g)		Dextrose; PN (%)		Dextrose; Suppl (%)		**2-4**	Enteral/IV fluid intake (ICR)	U/EBG	Bone/LFT	TG	AA	Growth
							
	std	max	std	max	std	max	std	max	PN type						

**8**	2.8	3.8	2.8	3.8	10	12	10	12	SCAMP	•	•				•

**9**	2.8	3.8	2.8	3.8	10	12	10	12	SCAMP	•	•	•			

**10**	2.8	3.8	2.8	3.8	10	12	10	12	SCAMP	•	•				

**11**	2.8	3.8	2.8	3.8	10	12	10	12	SCAMP	•	•	•			

**12**	2.8	3.8	2.8	3.8	10	12	10	12	SCAMP	•	•				

**13**	2.8	3.8	2.8	3.8	10	12	10	12	SCAMP	•	•	•			

**14**	2.8	3.8	2.8	3.8	10	12	10	12	SCAMP	•	•		•	•	

Legend: Daily flow chart summary of SCAMP nutrition study protocol including consent, randomisation, PN administration and data collection. Week 2 flow chart is repeated in week 3 and 4 to complete the 28 day intervention period. Day 29: Patient reverts to standard PN (if still on PN). All routine data collection stops apart from routine weekly growth data which continues until 36 weeks corrected for gestational age.

Abbreviations: stnd: standard PN (scNPN); max: scNPN_max_; ICR: intensive care record of daily fluid/nutrient/drug administration; U/E, BG: routine biochemical monitoring of plasma electrolytes, glucose, lactate and blood gases; Bone/LFT: routine biochemical monitoring of plasma bone and liver biochemistry; TG: triglyceride levels; AA amino acid levels.

### Intravenous/enteral nutrition, fluid and drug infusion data

The hourly volume of each component of the intravenous/enteral nutrition, fluid and drug infusions is captured on routine nursing charts. Each 24 hour period will start at the time of birth and data will be collected for 28 completed days after birth.

### Biochemical/nutritional monitoring

Biochemical and nutritional monitoring will follow the protocol outlined in the LWH NICU PN guidelines (incorporated in the study schedule summary in Appendix 1).

### Growth monitoring

Occipitofrontal head circumference, weight, mid-upper arm circumference and lower leg length will be measured after 7, 14, 21, 28 days and then weekly until 36 weeks CGA.

### Infection monitoring

Monitoring for infection will follow the protocol outlined in the LWH NICU guidelines for infection. Daily CRP, white cell count (and neutrophils) and platelet data will be recorded in medical record and transcribed to the appropriate CRF for 35 days from birth.

### Line complications

Vascular access device usage and location data will be recorded including extravasation episodes resulting in skin/tissue injury.

### Neurodevelopmental follow-up

Following discharge, infants of this gestation have routine, out-patient, neurodevelopmental follow-up. Parents of study infants will be approached again at 2 years CGA, to request a formal neurodevelopmental assessment (Bayley III). This will replace one of the routine OP assessments and take place in the home (where possible). It will be performed by a consultant in paediatric neurodisability.

### Blood sampling and processing

PN blood tests: Routine biochemical monitoring will take place in accordance with LWH PN guidelines (Appendix 1, section 2.1.4). All blood samples will be processed according to standard practice and sent to the laboratories at the Royal Liverpool Children's Hospital (Alder Hey).

## Methods: Statistical Analysis

The primary outcome of the study will be assessed by comparing the groups allocated to scNPN and scNPN_max_.

### Sample size

A sample size of 75 (assuming a survival rate of 80% of recruited infants) in each group will have 80% power to detect a difference between the means of the 2 scNPN groups for the outcome head growth velocity over the first 28 days after birth of 6 mm. This assumes that the common standard deviation (SD) is 12 mm and analysis is based on using a two group t-test with a 0.05 two-sided significance level. The value for the SD is based on data gathered during a randomised controlled trial of nutrition on this unit [17] and previous audit (Cooke unpublished data). This indicated that head growth velocity in the first 28 days was 24 mm/28d (SD12 mm). To maintain "normal" head growth (following the birth centile) a growth velocity of approximately 36 mm/28d is required at 24-28 weeks gestation. Head growth between birth and 28 days is has an approximately linear growth model (based on normal growth in utero). Thus the power calculation assumes that scNPN will achieve a mean growth velocity of 24 mm/28d (based on results from a nutritionally equivalent PN) and that the study has the power to detect an improvement in head growth to a mean growth velocity of ≥30 mm/28days using scNPN_max _assuming a common standard deviation of 12 mm.

### Analysis

A data analysis plan will be finalised when two thirds of participants have been recruited in order to allow the details of handling missing data to be based on experience with data collection. All analysis will be performed after data cleaning has been complete.

### Primary analysis

Primary analysis of the data will be by intention to treat, and will be done for all survivors. In order to test the hypothesis that the change in head circumference differs between the two groups while taking account of the clustering arising from multiple pregnancies, the primary outcome will be assessed using a general linear model.

### Secondary analysis to facilitate interpretation of the primary outcome

a) Developing a non-linear model of early head growth (if data analysis indicates this is required)

b) Longitudinal joint modelling of head growth and survival;

c) Longitudinal joint modelling of head growth and protein/calorie intake

d) per protocol analysis omitting babies that received PN other than that due under their allocation for more than 24 hours;

e) exploratory data analysis of how potential confounding variables are distributed between the two intervention groups

### Secondary analysis to characterise the trial

Exploratory data analysis will be used to describe the relationships between treatment allocation and:

a) growth measures expected to be concordant with the primary outcomes

b) efficiency of nutrient delivery

c) metabolic tolerance to each regimen

d) issues relating to the delivery of the nutritional regimens

e) major neonatal morbidity

f) neurodevelopmental outcome at 2 years

## Discussion

### Safety and adverse event reporting

Adverse events are relatively common in this patient group due to immaturity and to concomitant disease processes. Randomisation is essential for a comparison of safety among those receiving a study intervention compared to an appropriate comparator group. Routine clinical monitoring will be used to ensure that biochemical monitoring stays within limits defined within LWH clinical guidelines. Glucose and triglyceride monitoring have guidelines in place to allow PN to be adapted if abnormal levels arise. Abnormal amino acid profiles are discussed with a biochemist. These levels of PN macronutrients have been used in several previous studies without safety concerns.

Expected SAEs (Table 3) that are often observed during the course of care following birth at less than 30 weeks gestation before 36 PCA will be recorded on the specific CRF. All deaths or suspected overdoses will be reported to the Sponsor by the Chief Investigator within 24 hours using the SAE report form. All SAEs and deaths will be reported to and reviewed by the Sponsor and DMC at regular intervals throughout the trial. In order to examine whether the pattern of these events differs between the treatment groups, the incidence of these adverse events will be tabulated and presented to the DMC at intervals defined in the DMC Charter. Potential suspected unexpected serious adverse reactions (SUSARs) will be reported to the R & D department at LWH within 24 hours of the investigator becoming aware of them. The R&D Department will evaluate reported events according to severity, causality and expectedness according to the Sponsor's Standard Operating Procedures. SUSARs will be reported to MHRA/LREC within the statutory time-frames

**Table 3:** List of Expected Serious Adverse Events

Serious adverse event	**Estimated incidence **[17]
Death	20%
Necrotising enterocolitis (diagnostic radiological/surgical changes)	15%
Intracranial abnormality on cranial ultrasound scan	15%
(paraenchymal haemorrhage or focal white matter injury)	
Ventilator dependency (28 days) and/or oxygen dependency (36 weeks CGA)	65%
Patent ductus arteriosus medical or surgical management	25%
Retinal surgery for retinopathy of prematurity	5%
Pulmonary haemorrhage	5%
Infection ( positive blood culture with clinical signs)	65%
Persistent derangement of liver function tests (36 wks CGA)	10%
Serious extravasation injury (permanent scarring and/or/joint deformity)	<5%

CGA: corrected gestational age

### Trial Oversight

#### Data Monitoring and Ethics Committee (DMEC)

An independent Data Monitoring and Ethics Committee (DMEC) has been formed. During the period of recruitment, interim summaries of mortality and SAE will be supplied, in the strictest confidence, to the DMEC by the trial statistician. The DMEC has confirmed its terms of reference and frequency of meetings (approximately 6 monthly, depending on recruitment rate) in its first meeting, before the trial began. In the light of interim data and emerging evidence from other studies, the DMEC will inform the Trial Steering Committee if, in their view, there is proof beyond reasonable doubt that the data indicate that any part of the protocol is indicated or contraindicated either for all infants or for a particular subgroup of trial participants.

#### Trial Steering Committee (TSC)

A Trial Steering Committee has been formed to supervise the conduct of the study. The terms of reference were agreed in its first meeting (before the trial began). The TSC will meet (minimum frequency) within a month of all DMC meetings to consider their recommendations.

#### Study Timetable

Recruitment started in October 2009 following final protocol approval by ethics committee and the Medicines and Healthcare products Regulatory Agency (MHRA). It is anticipated recruitment will have completed in April 2012 allowing analysis of the primary outcome to be completed by December 2012. The last neurodevelopmental assessment would be completed in August 2014.

## Competing interests

The authors declare that they have no competing interests.

## Authors' contributions

CM, SH, IB developed the scNPN concept. CM and MAT formulated the study design with major contributions from SH and IB (pharmacy aspects), AH (statistical design and analysis), MT (growth and neurodevelopmental outcomes) and KM and PN (biochemical monitoring and analysis). All authors have read and approved the final manuscript.

## Pre-publication history

The pre-publication history for this paper can be accessed here:

http://www.biomedcentral.com/1471-2431/11/53/prepub

## References

[B1] Confidential Enquiry into Maternal and Child Health. Perinatal Mortality 2005: England, Wales and Northern Ireland 2007 CEMACH: London

[B2] Wood NS Marlow N Costeloe K Gibson AT Wilkinson AR Neurologic and developmental disability after extremely preterm birth. EPICure Study Group N Engl J Med 2000 343 378 84 10.1056/NEJM200008103430601 10933736

[B3] Clarke RH Wagner CL Merritt RJ Bloom BT Neu J Young TE Clark DA Nutrition in the intensive care unit: how do we reduce the incidence of extrauterine growth restriction? J Perinatol 2003 23 337 344 10.1038/sj.jp.7210937 12774145

[B4] Dusick AM Poindexter BB Ehrenkranz RA Lemons JA Growth failure in the preterm infant: can we catch up? Sem Perinatol 2003 27 302 10 10.1016/S0146-0005(03)00044-2 14510321

[B5] Ehrenkranz RA Dusick AM Vohr BR Wright LL Wrage LA Poole K Growth in the neonatal intensive care unit influences neurodevelopment and growth outcomes of extremely low birth weight infants Pediatrics 2006 117 1253 61 10.1542/peds.2005-1368 16585322

[B6] Ehrenkranz RA Younes N Lemons JA Fanaroff AA Donovan EF Wright LL Katsikiotis V Tyson JE Oh W Shankaran S Bauer CR Korones SB Stoll BJ Stevenson DK Papile L Longitudinal growth of hospitalized very low birthweight infants Pediatrics 1999 104 280 9 10.1542/peds.104.2.280 10429008

[B7] Clark RH Thomas P Peabody J Extrauterine growth restriction remains a serious problem in prematurely born neonates Pediatrics 2003 111 986 90 10.1542/peds.111.5.986 12728076

[B8] Wood NS Costeloe K Gibson AT Hennessy EM Marlow N Wilkinson AR The EPICure study growth and associated problems in children born at 25 weeks of gestational age or less Arch Dis Child Fetal Neonatal Ed 2003 88 F492 500 10.1136/fn.88.6.F492 14602697 PMC1763245

[B9] Cooke RW Lucas A Yudkin PLN Pryse-Davies J Head circumference as an index of brain weight in the fetus and newborn Early Hum Dev 1977 1 145 9 10.1016/0378-3782(77)90015-9 617306

[B10] Hack M Breslau N Very low birth weight infants: effects of brain growth during infancy on intelligence quotient at 3 years of age Pediatrics 1986 77 196 202 3945532

[B11] Hack M Breslau N Weissman B Arum D Klein N Borawski E Effect of very low birthweight and subnormal head size on cognitive abilities at school age N Eng J Med 1991 325 231 7 10.1056/NEJM199107253250403 2057024

[B12] Cooke RW Foulder-Hughes L Growth impairment in the very preterm and cognitive and motor performance at 7 years Arch Dis Child 2003 88 482 7 10.1136/adc.88.6.482 12765911 PMC1763118

[B13] Cooke RW Are there critical periods for brain growth in children born preterm? Arch Dis Child Fetal Neonatal Ed 2006 91 F17 20 16223756 10.1136/adc.2005.077438 PMC2672640

[B14] Cooke RJ Postnatal growth in preterm infants. In: Neonatal Nutrition and Metabolism. Thureen PJ, Hay WW (Eds) 2006 Cambridge University Press, Cambridge pp47 57

[B15] Berry MA Abrahamowicz M Usher RH Factors associated with growth of extremely premature infants during initial hospitalisation Pediatrics 1997 100 640 6 9310518

[B16] Embleton NE Pang N Cooke RJ Postnatal malnutrition and growth retardation: an inevitable consequence of current recommendations in preterm infants? Pediatrics 2001 107 270 3 10.1542/peds.107.2.270 11158457

[B17] Tan MJ Cooke RWI Improving head growth in very preterm infants - a randomized controlled trial I: neonatal outcomes Arch Dis Child Fetal Neonatal Ed 2008 93 F337 41 10.1136/adc.2007.124230 18252814

[B18] Donovan R Puppala B Angst D Coyle BW Outcome of early nutrition support in extremely low birth weight infants Nutr Clin Pract 2006 21 395 400 10.1177/0115426506021004395 16870808

[B19] Olsen IE Richardson DK Schmid CH Ausman LM Dwyer JT Intersite differences in weight growth velocity of extremely premature infants Pediatrics 2002 110 1125 1132 10.1542/peds.110.6.1125 12456909

[B20] Bloom BT Mulligan J Arnold C Ellis S Moffitt S Rivera A Kunamneni S Thomas P Clark RH Peabody J Improving growth of very low birth weight infants in the first 28 days Pediatrics 2003 112 8 14 10.1542/peds.112.1.8 12837860

[B21] Mason DG Puntis JW McCormick K Smith N Parenteral nutrition for neonates and children: a mixed bag Arch Dis Child 2010 10.1136/adc.2010.188557 20852278

[B22] Johnson JD Albritton WL Sunshine P Hyperammonaemia accompanying parenteral nutrition in preterm infants J Pediatr 1972 81 154 61 10.1016/S0022-3476(72)80395-0 4624623

[B23] te Braake FWJ van den Akker CHP Riedijk MA van Goudoever JB Parenteral amino acid and energy administration to premature infants in early life Sem Fetal Neonatal Med 2007 12 11 18 10.1016/j.siny.2006.10.002 17142119

[B24] te Braake FWJ van den Akker CHP Wattimena DJL Huijmans JG van Goudoever JB Amino acid administration to preterm infants directly after birth J Pediatr 2005 147 457 61 10.1016/j.jpeds.2005.05.038 16227030

[B25] Murdock N Crighton A Nelson LM Forsyth JS Low birthweight infants and parenteral nutrition immediately after birth. II Randomised study of biochemical tolerance of intravenous glucose, amino acids and lipid Arch Dis Child Fetal Neonatal Ed 1995 73 F8 12 10.1136/fn.73.1.F8 7552604 PMC2528370

[B26] Thureen PJ Melara D Fennessey PV Hay WW Effect of low versus high intravenous amino acid intake on very low birth weight infants in the early neonatal period Pediatr Res 2003 53 24 32 12508078 10.1203/00006450-200301000-00008

[B27] Ibrahim HM Jeroudi MA Baier RJ Dhanireddy R Krouskop RW Aggressive early total parenteral nutrition in low-birth-weight infants J Perinatol 2004 24 482 6 10.1038/sj.jp.7211114 15167885

[B28] Kotsopoulos K Benadiba-Torch A Cuddy A Safety and efficacy of early amino acids in preterm <28 weeks gestation: prospective observational comparison J Perinatol 2006 26 749 54 10.1038/sj.jp.7211611 17024139

[B29] Thureen PJ Anderson AH Baron KA Melara DL Hay WW Fennessey PV Protein balance in the first week of life in ventilated neonates receiving parenteral nutrition Am J Clin Nutr 1998 68 1128 35 9808233 10.1093/ajcn/68.5.1128

[B30] Jadhav P Parimi PS Kalhan SC Parenteral amino acid and metabolic acidosis in premature infants J Parenter Enteral Nutr 2007 31 278 283 10.1177/0148607107031004278 PMC1905854 17595435

[B31] Koletzko B Goulet O Hunt J Krohn K Shamir R Guidelines on Paediatric Parenteral Nutrition of the European Society of Paediatric Gastroenterology, Hepatology and Nutrition (ESPGHAN) and the European Society for Clinical Nutrition and Metabolism (ESPEN), Supported by the European Society for Paediatric Research (ESPR) J Paediatr Gastroentrol Nutr 2005 41 Suppl 2 S1 87 10.1097/01.mpg.0000181841.07090.f4 16254497

[B32] Kashyap S Schulze KF Thureen PJ, Hay WW Energy requirements and protein energy metabolism and balance in preterm and term infants Neonatal Nutrition and Metabolism 2006 Cambridge University Press, Cambridge 134 146

[B33] Thureen PJ Hay WW Intravenous nutrition and postnatal growth of the micropremie Clin Perinatol 2000 27 197 219 10.1016/S0095-5108(05)70014-2 10690572

[B34] Clark RH Chace DH Sptizer AR Effects of two different doses of amino acid s supplementation on growth and blood amino acid levels in premature infants admitted to the neonatal intensive care unit: a randomized controlled trial Pediatrics 2007 120 1286 96 10.1542/peds.2007-0545 18055678

[B35] Collins CT Gibson RA Miller J Carbohydrate intake is the main determinant of growth in infants born <33weeks gestation when protein intake is adequate Nutrition 2008 24 451 7 10.1016/j.nut.2008.01.014 18329853

[B36] Ogilvy-Stuart A Beardsall K Management of hyperglycaemia in the preterm infant Arch Dis Child Fetal Neonatal Ed 2010 95 F126 F131 10.1136/adc.2008.154716 20231218

[B37] Hans DM Pylipow M Long JD Thureen PJ Georgieff MK Nutritional practices in the neonatal intensive care unit: analysis of a 2006 neonatal nutrition survey Pediatrics 2009 123 51 57 10.1542/peds.2007-3644 19117860

[B38] Wilson DC Cairns P Halliday HL Reid M McClure G Dodge JA Randomised controlled trial of an aggressive nutrition regimen in sick very low birthweight infants Arch Dis Child Fetal Neonatal Ed 1997 77 F4 F11 10.1136/fn.77.1.F4 9279175 PMC1720665

[B39] Porcelli PJ Jr Sisk PM Increased parenteral amino acid administration to extremely low-birth-weight infants during early postnatal life J Pediatr Gastroenterol Nutr 2002 34 174 9 10.1097/00005176-200202000-00013 11840036

[B40] Dinerstein A Nieto RM Solana CL Perez GP Otheguy LE Larguia AM Early and aggressive nutritional strategy (parenteral and enteral) decreases postnatal growth failure in very low birth-weight infants J Perinatol 2006 26 436 42 10.1038/sj.jp.7211539 16801958

[B41] Poindexter BB Langer JC Dusick AM Ehrenkranz RA Early provision of parenteral amino acids in extremely low birth weight infants: relation to growth and neurodevlopmental outcome J Pediatr 2006 148 300 5 10.1016/j.jpeds.2005.10.038 16615955

[B42] Tan MJ Cooke RWI Abernethy L Improving head growth in very preterm infants - a randomized controlled trial II MRI and developmental outcomes in the first year Arch Dis Child Fetal Neonatal Ed 2008 93 F342 6 10.1136/adc.2007.124255 18285378

[B43] Stephens BE Walden RV Gargus RA Tucker R McKinley L Mance M Nye J Vohr BR First week protein and energy intakes are associated with 18-month developmental outcomes in extremely low birth weight infants Pediatrics 2009 123 1377 43 10.1542/peds.2008-2086 19403500

[B44] Stoltz Sjöström EKM Szymlek-Gay E Öhlund I Ahlsso F Norman M Engström E Hellström A Fellman V Olhager E serenius F Kallen K Domellof M Postnatal energy and protein deficits are associated with poor neonatal growth: preliminary results from a Swedish population-based study Pediatr Res 2010 68 suppl 1 64 5

[B45] Ahmed M Irwin S Tuthill DP Education and evidence are needed to improve neonatal parenteral nutrition practice J Parenter Enteral Nutr 2004 28 176 9 10.1177/0148607104028003176 15141411

[B46] Grover A Khashu M Mukherjee A Kairamkonda V Iatrogenic malnutrition in neonatal intensive care units: urgent need to modify practice J Parenter Enteral Nutr 2008 32 140 4 10.1177/0148607108314373 18407906

[B47] Ball PA Candy DCA Puntis JWL McNeish AS Portable bedside microcomputer system for management of parenteral nutrition in all age groups Arch Dis Child 1985 60 435 9 10.1136/adc.60.5.435 3925893 PMC1777300

[B48] Puago MA Nguyen HL Sheridan MJ Computerized PN ordering optimizes timely nutrition therapy in a neonatal intensive care unit J Am Diet Assoc 1997 97 258 261 10.1016/S0002-8223(97)00067-9 9060941

[B49] Eleni-dit-Trolli S Kermorvant-Duchemin E Huon C Early individualized parenteral nutrition for preterm infants Arch Dis Child Fetal Neonatal Ed 2009 94 F152 3 10.1136/adc.2007.136333 18838470

[B50] Riskin A Shiff Y Shamir R Parenteral nutrition in neonatology - to standardize or individualize? IMAJ 2006 8 641 5 17058418

[B51] Fusch C Bauer K Bohles HJ Jochum F Koletzko B Krawinkel M Krohn K Muhlebach S Neonatology/paediatrics - guidelines on parenteral nutrition, Chapter 13 German Medical Science 2009 7 1 23 10.3205/000074 PMC2795370 20049070

[B52] Smolkin T Diab G Shohat I Jubran H Blazer S Rozen GS Makhoul IR Standardized versus individualized parenteral nutrition in very low birth weight infants: a comparative study Neonatology 2010 98 170 8 10.1159/000282174 20234142

[B53] Hartwig SC Gardner DK Use of standardized Total Parenteral Nutrition solutions for preterm neonates Am J Hosp Pharm 1989 46 993 5 2499193

[B54] Dice JE Burckart GJ Woo JT Helms RA Standardized versus pharmacist-monitored individualised parenteral nutrition in low-birth-weight infants Am J Hosp Pharm 1981 38 1487 9 6794364

[B55] Beecroft C Martin H Puntis JWL How often do parenteral nutrition prescription for the newborn need to be individualised? Clin Nutr 1999 18 83 5 10.1016/S0261-5614(99)80056-9 10459069

[B56] Keady S Morgan C Ozzard A Chauhan B Effect of a standard neonatal aqueous parenteral nutrition formulation on aseptic unit capacity planning European e-Journal Clinical Nutrition and Metabolism 2010 5 e14 e17 10.1016/j.eclnm.2009.10.009

[B57] Yeung MY Smyth JP Maheshwari R Shah S Evaluation of standardized versus individualized total parenteral nutrition regime for neonates less than 33 weeks gestation J Paediatr Child Health 2003 39 613 7 10.1046/j.1440-1754.2003.00246.x 14629529

[B58] Lenclen R Crauste-Manciet S Narcy P Boukhouna S Geffray A Guerrault MN Bordet F Brossard D Assessment of implementation of a standardized parenteral nutrition formulation for early nutritional support of very preterm infants Eur J Pediatr 2006 165 512 8 10.1007/s00431-006-0124-1 16622662

[B59] Skouroliakou M Koutri K Stathopoulou M Vourvouhaki E Giannopoulou I Gounaris A Comparison of two types of TPN prescription methods in preterm neonates Pharm World Sci 2009 31 202 8 10.1007/s11096-009-9281-4 19169838

[B60] Morgan C Badhawi I Grime C Herwitker S Improving early protein intake in very preterm infants using a standardised concentrated neonatal parenteral nutrition formulation European e-Journal Clinical Nutrition and Metabolism 2009 4 e324 e328 10.1016/j.eclnm.2009.09.004

